# Effects of Organic Fertilizer Type and Application Rate on Soil–Microbe Interactions, Yield, and Quality of Greenhouse Tomato

**DOI:** 10.3390/plants14213333

**Published:** 2025-10-31

**Authors:** Jingshi Lu, Xiaoming Zhang, Yingtong Mu, Jiahui Gao, Fengyan Yi, Ping Wang, Doudou Jin, Fang Tang, Wenqiang Fan

**Affiliations:** 1 Key Laboratory of Grassland Resources, College of Grassland Science, Mongolian and Chinese Medicinal Plant Germplasm Breeding Engineering Technology Research Center, Inner Mongolia Agricultural University, Ministry of Education, Hohhot 010011, China; jingshilu37@gmail.com (J.L.); bagenna123@aliyun.com (X.Z.); myt100862@outlook.com (Y.M.); 19819262059@163.com (P.W.); chinajddimau@163.com (D.J.); 2 Inner Mongolia Forestry and Grassland Monitoring and Planning Institute, Hohhot 010011, China; 13474710702@163.com; 3 Inner Mongolia Academy of Agricultural and Animal Husbandry Sciences, Hohhot 010031, China; yifengyanonly88@126.com

**Keywords:** rhizosphere microbiome, microbial interactions, structural equation modeling (PLS-PM), photosynthetic traits, soil fertility

## Abstract

Soil nutrient imbalance and the decline of microbial diversity threaten the long-term sustainability of crop production in intensive agriculture. Organic fertilizers provide a promising means to improve soil–microbe–plant interactions, yet the combined effects of fertilizer type and application rate on soil function and crop productivity remain insufficiently understood. In this study, we investigated the agronomic and ecological responses of greenhouse tomato (*Solanum lycopersicum* L.) to three organic fertilizers—bone calcium fertilizer (BCF), bone mud fertilizer (BMF), and bio-organic fertilizer (BOF)—each applied at four rates (7500, 15,000, 30,000, and 45,000 kg·ha^−1^). The highest tested BOF rate (45,000 kg·ha^−1^) significantly increased net photosynthesis by 29.5%, stomatal conductance by 50.0%, and fruit yield by 40.8% compared with the unfertilized control. It also enhanced soil organic matter by 42.6% and total nitrogen by 82.0%, while increasing the relative abundance of Proteobacteria, a phylum closely associated with nutrient cycling and plant growth promotion. Network and path modeling revealed that changes in microbial diversity were positively associated with improved soil properties, which were subsequently linked to higher photosynthetic efficiency and yield formation, suggesting a potential microbiome-mediated pathway from fertilization to productivity. These effects were statistically consistent across measured endpoints. Our findings highlight that optimizing both the type and rate of organic fertilizer—particularly bio-organic fertilizer under greenhouse conditions—can enhance soil fertility, microbial function, and crop yield simultaneously. This study provides an evidence-based framework for precision fertilization strategies aimed at improving agroecosystem resilience and advancing sustainable tomato production.

## 1. Introduction

Tomato (*Solanum lycopersicum* L.) is a globally important horticultural crop with substantial economic significance in both fresh markets and processing industries [1,2,3]. In China, its production is increasingly concentrated in greenhouse systems, where advanced solar greenhouse technology allows precise control of light, temperature, and humidity, thereby improving yield and fruit quality [4,5]. However, intensive greenhouse-based farming still faces persistent challenges, including land-use constraints, disease pressure, and declining resource-use efficiency [6,7,8]. Overreliance on chemical fertilizers further exacerbates soil degradation, nutrient imbalances, and reduced microbial activity, ultimately threatening the sustainability of high-input agricultural systems [9]. These issues underscore the urgent need to develop fertilization strategies that can sustain soil fertility, enhance microbial function, and support stable crop productivity under intensive greenhouse conditions.

Organic fertilizers (OFs), derived from renewable biological materials, have emerged as pivotal tools in the transition toward sustainable agriculture. Their multifunctional benefits—enhancing soil fertility, improving structure, increasing organic matter content, and stimulating microbial activity—position them as ecologically sound alternatives to chemical fertilizers [10,11]. Judicious application of OFs can facilitate long-term nutrient release, promote microbial-mediated nutrient cycling, and enhance crop productivity and resilience. However, limitations such as variable quality, relatively high costs, and inconsistent field performance hinder their broader adoption in high-efficiency protected cultivation systems. Furthermore, current studies often focus on single fertilizer types or specific application rates, lacking a systematic comparison of different organic fertilizers and their interactive effects on the soil–microbe–plant continuum [12,13].

Soil microbial communities are integral to nutrient dynamics, soil health, and plant performance. Fertilization influences not only soil physicochemical properties but also microbial structure and function, mediating nutrient availability and plant growth through belowground–aboveground feedbacks. In turn, plant roots exude metabolites that shape microbial community composition, forming a tightly coupled tripartite system of soil–microbe–plant interactions [14,15,16,17]. Understanding the mechanisms by which organic fertilizers modulate this complex system is essential for designing optimized nutrient management strategies.

Despite growing interest in organic fertilization, how fertilizer type and application rate jointly shape the soil–microbe–plant system of greenhouse tomato remains insufficiently resolved, particularly with respect to integrative links among soil properties, rhizosphere microbiota, and crop performance. Here, we set out to determine how three organic fertilizer types—bio-organic fertilizer (BOF), bone meal fertilizer (BMF), and bone calcium fertilizer (BCF)—applied at 7500, 15,000, 30,000, and 45,000 kg·ha^−1^ affect plant performance (growth, yield, fruit quality) together with concurrent changes in soil physicochemical properties and the rhizosphere microbiome (alpha diversity and community composition). We further examined soil–microbe linkages using co-occurrence network analysis and Mantel tests and evaluated indirect pathways connecting fertilization, soil and microbial attributes, and crop performance via partial least squares path modeling (PLS-PM) to identify agronomically suitable application ranges under greenhouse conditions. Based on prior evidence, we hypothesized that fertilizer type and rate would be associated with shifts in soil resource status and rhizosphere microbial diversity/structure, which would, in turn, be linked to variation in photosynthetic traits and yield.

## 2. Results

### 2.1. Growth Characteristics Analysis

#### 2.1.1. Response of Tomato Photosynthetic Physiology to Different Fertilization Regimes

Net photosynthetic rate (Pn), stomatal conductance (gs), and transpiration rate (Tr) were measured under three organic fertilizer treatments—bone calcium fertilizer (BCF), bone mud fertilizer (BMF), and bio-organic fertilizer (BOF)—each applied at four dosage levels, along with an unfertilized control. Across all developmental stages, fertilization significantly increased Pn, gs, and Tr relative to the control (*p* < 0.01; Table S1). Percent changes reported below are defined relative to the concurrent control at the same sampling stage.

During the flowering and fruit-setting stages, net photosynthetic rate (Pn) under BOF4 reached 34.9 μmol CO_2_·m^−2^·s^−1^ (+29.5% vs. control at the same stage), exceeding BCF4 (+25.5%) and BMF4 (+18.8%). A similar pattern was observed at the fruit-expansion stage, where CO_2_ assimilation under BOF4 consistently surpassed the other treatments. By contrast, low-dose treatments (BCF1 and BMF1) produced only modest gains in Pn (+12.0% and +7.4%, respectively), indicating that higher-rate BOF most strongly enhances carbon-assimilation capacity under greenhouse conditions (Table S1).

Stomatal conductance (gs) likewise peaked under BOF4, reaching 0.50 mol H_2_O·m^−2^·s^−1^ (+50.0% vs. control at the same stage), with BCF4 and BMF4 increasing by +42.0% and +37.0%, respectively. The elevated gs under BOF4 suggests enhanced stomatal opening and CO_2_ diffusion into the mesophyll, thereby supporting sustained photosynthetic activity (Table S1).

Transpiration rate (Tr) was also enhanced by fertilization; at flowering, BOF4 recorded 19.2 mmol H_2_O·m^−2^·s^−1^ (+24.5% vs. control at the same stage), higher than BCF4 (+17.2%) and BMF4 (+10.4%). The higher Tr under BOF4 likely contributed to thermal regulation and improved gas exchange within the greenhouse microclimate, supporting overall photosynthetic performance and stress tolerance (Table S1).

Taken together, organic fertilization markedly alters leaf gas-exchange dynamics in greenhouse tomatoes. Among all treatments, high-dose BOF (BOF4) produced the most pronounced improvements in photosynthetic capacity, underscoring its superior efficacy in optimizing physiological processes that underpin growth and productivity.

#### 2.1.2. Effect of Different Fertilization Treatments on Plant Height and Stem Diameter of Greenhouse Tomatoes

Organic fertilization significantly enhanced tomato vegetative growth, with both plant height and stem diameter showing consistent, dose-dependent increases across treatments (Figure S1a,b). High-rate bio-organic fertilizer (BOF4) exhibited the greatest effect, with final plant height and stem diameter at 70 days post-transplanting reaching 136.9 cm and 14.5 mm, respectively, both markedly higher than other treatments and the unfertilized control (*p* < 0.01). Lower application rates produced only minor improvements relative to the control. These findings indicate that BOF, particularly at higher rates, can sustain structural growth and vigor throughout the cultivation period.

### 2.2. Tomato Yield and Quality Analysis

At the onset of the harvest stage, single-plant tomato yields were measured across treatments. Fertilization significantly increased individual plant yield, with a dose-dependent trend observed. Among the fertilizer types, the yield-promoting effects followed the order: BOF > BCF > BMF. Compared with the control, organic fertilization led to yield increases ranging from 4.3% to 40.8%. The BOF4 treatment recorded the highest single-plant yield at 8.2 kg·plant^−1^, representing a 40.8% improvement over the control. BCF4 and BMF4 achieved yields of 8.10 kg and 8.1 kg per plant, respectively, showing increases of 38.9% and 38.4%, and were significantly higher than most other treatments, indicating the yield-enhancing potential of high-dose organic fertilizer applications (Table S2).

Subsequent total yield estimations were extrapolated to a per-hectare basis. The highest yield was observed in the BOF4 treatment, forming a high-yield cluster alongside BMF4, BCF3, and BCF4, with no statistically significant differences among them. Treatments such as BCF2, BOF2, and BOF3 constituted the medium-yield group, while BCF1, BOF1, BMF2, and BMF3 comprised the low-yield group. BMF1 and the control consistently showed the lowest yields. These results were consistent with single-plant data, further reinforcing the superiority of BOF—especially BOF4—as the most effective in yield enhancement, followed by BCF, with BMF exhibiting relatively modest effects (Table S2).

In terms of fruit quality, as assessed by soluble sugar content (SSC, °Brix), fertilization treatments significantly influenced sugar accumulation. Treatments achieving > 7.0 °Brix sugar content included BOF4, BCF4, and BMF4, representing 23% of total treatments and meeting the criteria for high-sweetness classification. Treatments with sugar contents between 6.5 °Brix and 7.0 °Brix—considered “good sweetness”—included BCF2, BCF3, BMF1, BMF3, and BOF3, accounting for 38.5%. Treatments such as BCF1, BMF2, BOF1, and BOF2 had sugar contents between 6.0 °Brix and 6.5 °Brix, while the control group fell below 6.0 °Brix, classifying as low sweetness. Notably, BCF4 ranked second only to BOF4 in sugar content and was significantly higher than most other treatments, underscoring its quality-enhancing capacity. BOF3 ranked third and was also significantly superior to the majority of treatments. No significant differences were observed among BCF2, BCF3, BMF1, and BMF3, nor among BCF1, BMF2, BOF1, and BOF2. Overall, BOF demonstrated superior efficacy in improving both yield and fruit quality, followed by BCF, while BMF showed relatively weaker performance (Table S2).

### 2.3. Soil Physicochemical Properties Analysis

#### 2.3.1. Changes in Soil Moisture Content

Fertilization treatments significantly influenced soil moisture content (Table S3). At 56 days post-planting, BOF1 treatment showed the highest moisture (48.1%, *p* < 0.01), while BOF4 had the lowest (41.4%). By day 70, BCF2 and BMF3 treatments had significantly higher moisture (32.8% and 32.2%) than others, while BOF4 remained lowest (30.8%). These results suggest that moderate doses of BCF and BMF contribute to soil moisture retention, while higher BOF doses may disrupt water retention.

#### 2.3.2. Changes in Soil pH

Soil pH exhibited a general decline with increasing fertilizer application across all treatments, reflecting the acidifying effects of organic fertilizers (Table S4, 70 DAP). The pH values in BCF1, BMF2, and BOF1 were significantly higher than those in high-dose treatments such as BCF4 and BOF4, yet still lower than the unfertilized control (pH = 7.15). These results indicate that high-dose organic fertilization may lead to notable soil acidification, which could potentially alter nutrient availability and microbial activity.

#### 2.3.3. Changes in Soil Nutrients

Organic fertilizer treatments markedly increased the contents of soil organic matter (OM), total nitrogen (TN), available phosphorus (AP), and available potassium (AK), with concentrations rising proportionally to fertilizer dosage. The BOF4 treatment group showed the highest OM content (30.9 g·kg^−1^), representing a 42.6% increase over the control group, highlighting the superior organic matter enrichment capacity of BOF. BCF and BMF treatments also enhanced OM levels, albeit to a lesser extent (Table S4).

Total nitrogen content was significantly elevated under all fertilization treatments. BOF4 achieved the highest TN level (0.3 mg·kg^−1^), 82% higher than the control, indicating BOF’s superior nitrogen-supplying capacity, likely attributed to its stable N-release formulation.

Available phosphorus also exhibited a clear upward trend with increasing fertilizer levels. High-dose treatments (BCF4, BMF4, BOF3, BOF4) all exceeded 150 mg·kg^−1^ in AP, a range considered agronomically optimal for tomato root uptake and stress resistance enhancement.

Available potassium was elevated across all treatments, with levels ranging from 200 to 400 mg·kg^−1^. BOF4 recorded the highest AK (287.7 mg·kg^−1^), slightly ahead of BCF4 (285.6 mg·kg^−1^), indicating that high-dose fertilization effectively met potassium demand during critical growth periods (Table S4).

### 2.4. Soil Microbial Community Analysis

#### 2.4.1. Alpha Diversity

To aid interpretation, we briefly define the diversity metrics used here: OTU richness/Chao1 index represents species richness (number of taxa; Chao1 is a richness estimator accounting for rare taxa), while Shannon and Simpson indices describe alpha diversity, with Shannon emphasizing both richness and evenness and Simpson emphasizing evenness/dominance (higher Simpson = more even). Illumina NovaSeq sequencing produced a total of 5,218,818 raw reads from 39 soil samples, and after stringent quality control, 4,818,485 high-quality sequences were retained. The Q20 values ranged from 99.3% to 99.5%, indicating high sequencing accuracy and reliability. The rarefaction curves reached a plateau, suggesting that the sequencing depth was sufficient to capture the majority of microbial diversity and that the data were adequate for downstream analyses (Table S4).

**Figure 1:**
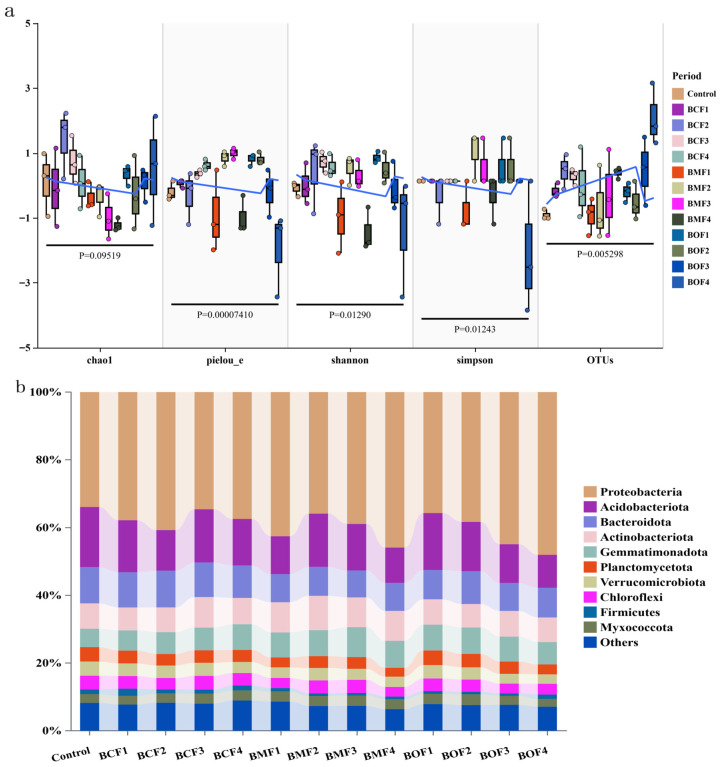

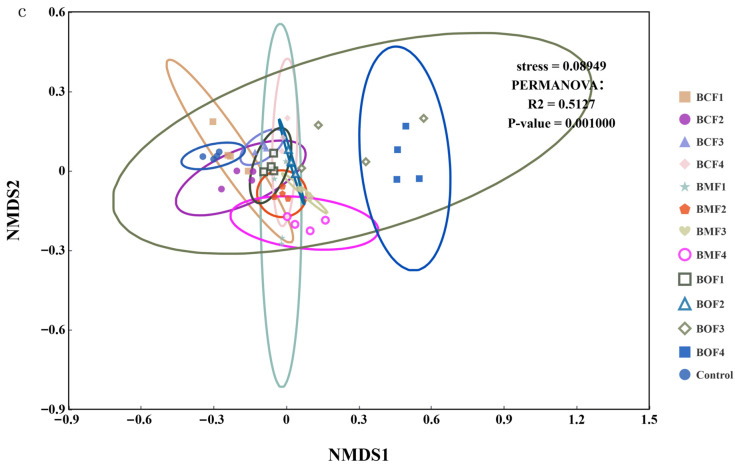
Effects of organic fertilizer treatments on soil bacterial communities. (**a**) Alpha-diversity indices—Chao1, Shannon, Simpson (dimensionless); *x*-axis: treatment; *y*-axis: index value. Boxes denote the interquartile range (IQR) with the center line indicating the median; whiskers extend to 1.5 × IQR; dots represent individual samples (jittered for visibility). Horizontal bars with accompanying *p*-values summarize overall treatment effects; the blue line indicates the fitted trend across treatments. Colors correspond to treatments as shown in the legend. Alpha-diversity indices are dimensionless. Statistical significance was evaluated at *p* < 0.05. (**b**) Relative abundance (%) of the top 10 bacterial phyla; *x*-axis: phylum; *y*-axis: relative abundance (%); bars grouped by treatment. (**c**) NMDS ordination of community composition based on Bray–Curtis distances; axes: NMDS1 and NMDS2 (dimensionless); PERMANOVA reports R^2^ and *p*. Abbreviations: BCF = Bone calcium organic Fertilizer; BMF = Bone mud organic Fertilizer; BOF = Bio-organic fertilizer; NMDS = non-metric multidimensional scaling; PERMANOVA = permutational multivariate analysis of variance.

Alpha diversity analysis revealed that the application of BOF, particularly under the BOF4 treatment, significantly enhanced the operational taxonomic unit (OTU) richness and Chao1 index of the soil microbial community compared to the control. This indicates a marked improvement in microbial species richness. BCF4 ranked second in terms of richness indices. Furthermore, the Shannon index demonstrated that BCF3 and BCF4 treatments notably increased microbial diversity, with improvements of 2.58–2.77% relative to the control. In contrast, the Simpson index showed that only the BOF1 treatment significantly improved community evenness, while other treatments exhibited no significant differences compared to the control. These results suggest that organic fertilizer application primarily enhanced microbial richness while exerting limited effects on community evenness (Figure 1a).

#### 2.4.2. Major Species Composition and Abundance Analysis

At the phylum level, the dominant bacterial taxa across all treatment groups were Proteobacteria, Acidobacteriota, Bacteroidota, and Actinobacteriota, which are widely recognized as core microbial constituents involved in soil nutrient cycling and organic matter turnover. Notably, the application of high-dose BOF significantly enriched the abundance of Proteobacteria, with its relative abundance reaching 48.0% in the BOF4 treatment, compared to 33.9% in the unfertilized control. This represents a substantial increase, suggesting that BOF promotes the proliferation of copiotrophic and functionally beneficial taxa. Similarly, BMF4 and BOF3 treatments also led to relatively higher abundances of Proteobacteria, reaching 45.9% and 44.9%, respectively, further underscoring the fertilization-dependent shifts in microbial community composition (Figure 1b).

#### 2.4.3. NMDS + PERMANOVA Analysis

To interpret beta-diversity patterns mechanistically and test them statistically, we combined non-metric multidimensional scaling (NMDS)—which visualizes multivariate differences in community composition—with permutational multivariate analysis of variance (PERMANOVA)—which formally tests whether between-group differences are significant on the same dissimilarity matrix (Bray–Curtis). In this framework, NMDS stress quantifies the goodness-of-fit of the ordination (lower values = better), whereas PERMANOVA R^2^ indicates the variance explained by treatment (effect size) and *p* provides permutation-based significance.

The NMDS ordination revealed distinct separations among treatment groups, indicating that both fertilizer type (BCF, BMF, BOF) and application rate exerted substantial impacts on microbial assemblages. Spatial proximity of sample points reflected compositional similarity, with the BOF4 treatment (blue diamonds) forming a tightly clustered group that was clearly segregated from others along the NMDS axes, suggesting a pronounced restructuring of the microbiome under high BOF input (Figure 1c). Conversely, treatments such as BMF1 exhibited dispersed sample distributions, implying elevated intra-treatment heterogeneity and a less deterministic response of microbial communities.

The PERMANOVA analysis statistically validated the observed patterns, revealing a significant effect of fertilization treatments on community structure (R^2^ = 0.5127, *p* = 0.001), with treatments explaining 51.3% of the observed compositional variance. Furthermore, the low NMDS stress value (0.0895 < 0.1) indicates high fidelity of dimensional reduction, substantiating the reliability of the ordination.

Collectively, these results demonstrate that fertilization significantly modulated the soil microbial community composition, with BOF4 exerting the most substantial influence. Differential community assembly across treatments is likely driven by alterations in soil physicochemical conditions, which mediate microbial niche selection and functional processes, ultimately influencing nutrient cycling and plant performance.

### 2.5. Mantel Test Analysis

The Mantel test further validated the intricate associations among soil microbial communities, physicochemical soil parameters, and plant phenotypic traits. A significant negative correlation between *Vicinamibacteraceae* and *Pseudomonas* (r = −0.426, *p* = 0.0069) suggests potential antagonism in resource acquisition or ecological niche overlap, which could limit functional redundancy within the rhizosphere. Conversely, a strong positive correlation between *Acinetobacter* and *Pseudomonas* (r = 0.606, *p* < 0.001) indicates possible functional effects, as both genera are known to participate in organic matter degradation and nutrient cycling, thereby supporting plant nutrient acquisition. Additionally, *Vicinamibacteraceae* exhibited a highly significant positive association with *RB4*1 (r = 0.798, *p* < 0.001), implying shared ecological strategies that may stabilize soil microbial communities under similar environmental conditions. The significant positive correlation between *Lysobacter* and *Acinetobacter* (r = 0.472, *p* = 0.0024) points toward potential cooperative roles in suppressing pathogens and enhancing soil enzymatic activity.

**Figure 2:**
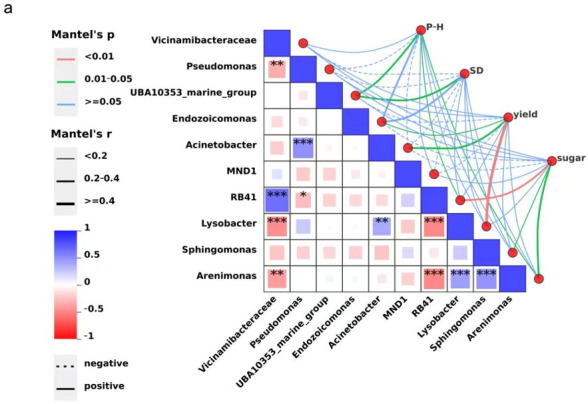

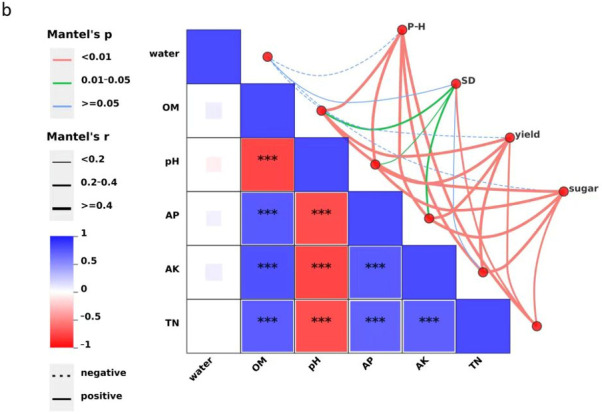
Mantel test analysis showing key drivers of microbial community distribution. (**a**) Correlations between the top 10 dominant bacterial genera (relative abundance) and plant traits (P–H, plant height; SD, stem diameter). (**b**) Correlations between soil physicochemical properties and plant traits (OM, organic matter; TN, total nitrogen; AP, available P (Olsen-P); AK, available K). Asterisks indicate significance levels: “*” *p* < 0.05; “**” *p* < 0.01; “***” *p* < 0.001.

Microbe–plant trait correlations revealed functionally relevant associations. Plant height was significantly and positively correlated with UBA10353_marine_group, *RB41*, and *Acinetobacter* (*p* < 0.05), all of which are potentially involved in nutrient mobilization and phytohormone production. Stem diameter was significantly associated only with UBA10353_marine_group (*p* < 0.05), suggesting its role in enhancing structural growth via improved nutrient uptake efficiency. Yield displayed significant positive correlations with *Endozoicomonas* and Acinetobacter and a very strong correlation with *Lysobacter* (*p* < 0.01), taxa that may contribute to improved nutrient assimilation and biocontrol functions. Soluble sugar content was significantly positively associated with *Sphingomonas* and *Acinetobacter* (*p* < 0.05) and highly significantly with RB41 (*p* < 0.01), suggesting that these taxa could facilitate carbohydrate biosynthesis through metabolic modulation of carbon fluxes (Figure 2a).

Similarly, soil physicochemical variables were closely linked to plant growth and fruit quality. Plant height was very significantly positively correlated with soil organic matter (OM), pH, available phosphorus (AP), available potassium (AK), and total nitrogen (TN) (*p* < 0.01). Stem diameter was also very significantly correlated with TN (*p* < 0.01) and significantly with OM, pH, and AP (*p* < 0.05). Both yield and sugar content exhibited very strong positive associations with OM, pH, AP, AK, and TN (*p* < 0.01) (Figure 2b). OM, as a core indicator of soil health, likely enhances plant productivity by improving nutrient availability, enriching beneficial microbial populations, and buffering soil acidity. In contrast, the observed negative correlation between pH and AP availability suggests that elevated pH may reduce phosphorus solubility, thereby constraining crop development. Soil moisture content exhibited a weak negative correlation with plant height, yield, and sugar content, potentially reflecting the complex interactions of water with other abiotic and biotic factors in the greenhouse environment.

### 2.6. Co-Occurrence Network Analysis

**Figure 3:**
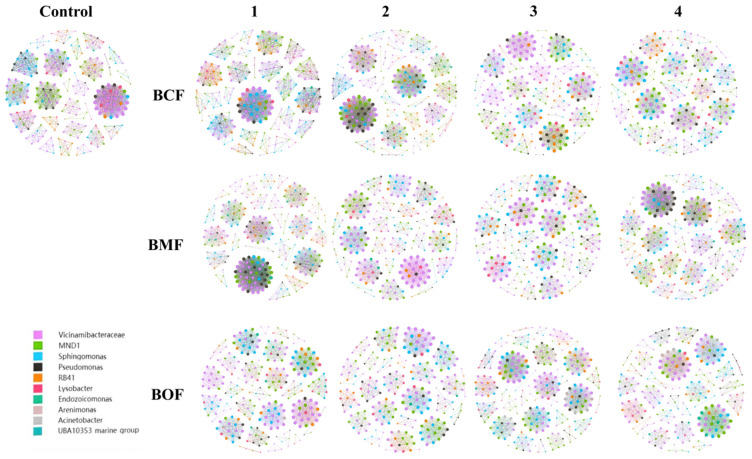
Co-occurrence network analysis of soil microorganisms under different organic fertilizer treatments and application rates. Panels are arranged by fertilizer (rows: BCF, BMF, BOF) and application rate (columns 1–4: 1 = 7500, 2 = 15,000, 3 = 30,000, 4 = 45,000 kg·ha^−1^), with the unfertilized control shown at far left. Nodes (circles/dots) represent bacterial genera; node color denotes taxonomic affiliation (see legend), and node size is proportional to degree (number of connections). Edges (lines) indicate significant co-occurrence relationships between genera (correlation criteria described in Section 2.6). Abbreviations: BCF, bone calcium organic fertilizer; BMF, bone mud organic fertilizer; BOF, bio-organic fertilizer.

The co-occurrence network analysis revealed that both fertilizer type and application concentration exerted significant impacts on the topological structure, connectivity, and modular organization of the soil microbial community (Figure 3). Compared with the untreated control (Control), fertilized treatments exhibited pronounced differences in network density, node and edge numbers, and clustering coefficients, suggesting that fertilization substantially modulates microbial community interactions (Table S6).

Low-concentration treatments (BCF1, BMF1, BOF1) tended to maintain higher network stability and promote cooperative interactions within the microbial community, as indicated by elevated clustering coefficients (e.g., BOF1: 0.92). In contrast, high-concentration treatments (BCF4, BMF4, BOF4) resulted in increased network complexity, exemplified by a notable rise in node number under BMF4 treatment, potentially reflecting intensified microbial competition and niche differentiation.

Fertilizer type also differentially influenced network properties. BCF series fertilizers, particularly BCF2, significantly increased network density (0.0605) and average degree (16.34), indicating an enhancement in inter-microbial connectivity. Interestingly, BMF1 exhibited the highest overall network density (0.0612), suggesting that low concentrations of BMF strongly promote microbial interaction networks. However, the clustering coefficient declined under high-concentration BMF4 treatment, implying potential disruption of cooperative structures due to competitive stress.

The BOF series fertilizers demonstrated a consistent positive effect on network cohesion. Specifically, BOF2 exhibited the highest clustering coefficient (0.92), reflecting enhanced cooperative interactions and potential community stabilization under this treatment.

Modularity analysis further highlighted the effects of fertilization on microbial functional organization. BMF1 treatment yielded a relatively low modularity index (0.74), suggesting limited compartmentalization and functional differentiation. In contrast, BOF2 exhibited the highest modularity (0.92), indicating enhanced functional partitioning likely driven by the selective enrichment of specific microbial guilds.

Collectively, these findings demonstrate that fertilization profoundly alters microbial network architecture, thereby influencing ecological functionality and stability. Low-concentration fertilization tends to sustain cooperative interactions and network stability, whereas high-concentration inputs may intensify competitive dynamics and restructure microbial niche distribution. Among the tested fertilizers, BOF series treatments exerted the most substantial influence in promoting microbial network stability and modular complexity, underscoring their potential to enhance soil microbial ecosystem resilience through selective facilitation of functional microbial groups.

### 2.7. PLS-PM Analysis

Partial Least Squares Path Modeling (PLS-PM) was employed to unravel the complex interactions among microbial diversity, soil physicochemical properties, plant growth, photosynthetic activity, and yield quality, thereby elucidating potential mechanisms underlying soil–microbe–plant interactions. The model demonstrated strong overall explanatory power, with a goodness-of-fit (GoF) value of 0.6503.

Path analysis revealed that microbial diversity exerted the strongest direct effect on soil properties (path coefficient β = 0.7287, *p* < 0.0001), emphasizing the foundational role of microbial communities in regulating soil nutrient availability, structure, and overall environmental quality. In turn, soil properties had significant positive effects on both plant growth (β = 0.4717, *p* = 0.0089) and photosynthetic efficiency (β = 0.7319, *p* < 0.0001), indicating that improved soil quality facilitates not only vegetative development but also physiological performance. Additionally, plant growth positively influenced photosynthesis (β = 0.3869, *p* = 1.58 × 10^−4^), suggesting a reinforcing relationship between biomass accumulation and resource assimilation capacity.

In terms of yield formation, soil properties had the most pronounced direct impact on yield quality (β = 0.5205, *p* = 0.0109), followed by microbial diversity (β = 0.2783, *p* = 0.0275), underscoring the central role of soil–microbe interactions in determining crop productivity. Furthermore, microbial diversity also had a strong indirect effect on yield quality through soil improvement pathways (indirect β = 0.4813), highlighting its multifaceted contribution to agroecosystem function. Conversely, photosynthesis exhibited a weak and statistically non-significant direct effect on yield quality (β = 0.1842, *p* = 0.3706), and plant growth showed no significant direct association (β = −0.0207, *p* = 0.8787), indicating that aboveground traits alone are insufficient to explain yield variability under the tested conditions.

**Figure 4:**
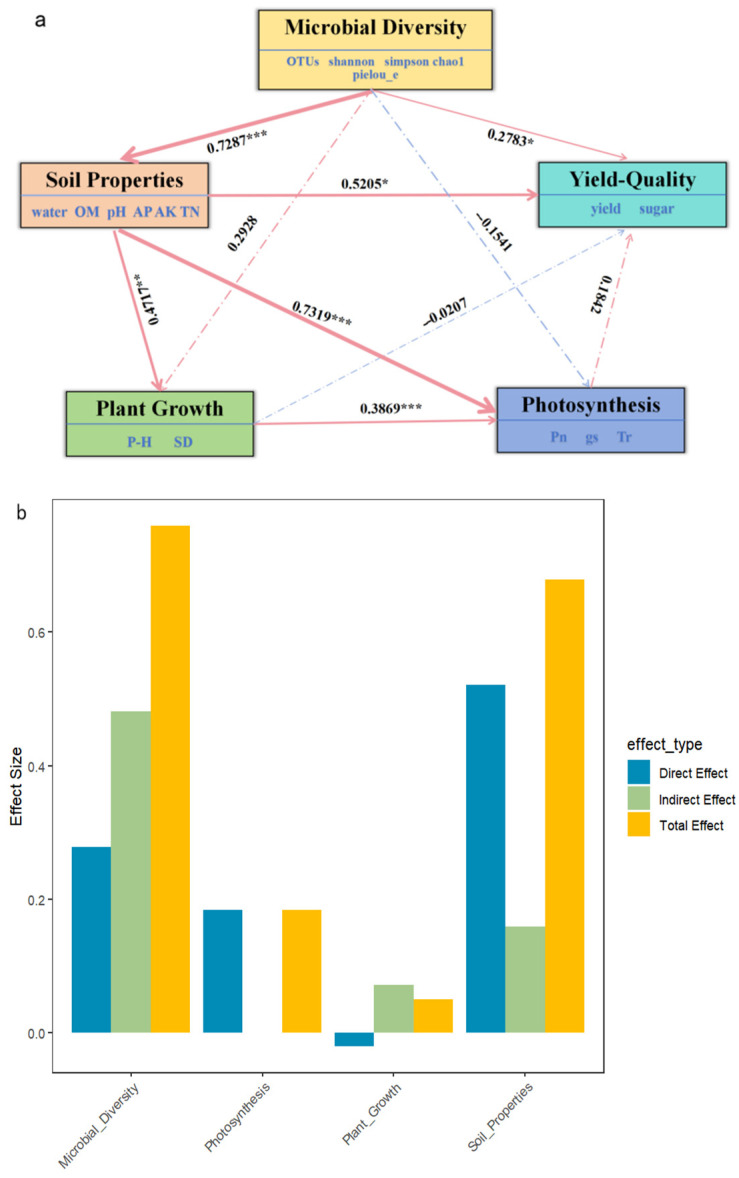
PLS-PM analysis (**a**) partial least squares path modeling (PLS-PM) analysis showing the key path relationships between microbial diversity, soil properties, plant growth, photosynthesis, and yield quality. Arrows represent direct effects between variables, with red indicating positive correlations and blue indicating negative correlations. ** indicates significance levels (* *p* < 0.05, ** *p* < 0.01, *** *p* < 0.001). (**b**) Bar plot of effect sizes from PLS-PM analysis. It displays the sizes of direct effects, indirect effects, and total effects. Different colors represent different effect types: blue for direct effects, green for indirect effects, and yellow for total effects.

The model’s coefficients of determination (R^2^) confirmed its strong explanatory capacity across system components. Microbial diversity explained 53.1% of the variance in soil properties (R^2^ = 0.531); soil conditions accounted for 51.0% of the variance in plant growth (R^2^ = 0.510); photosynthesis was largely driven by soil and plant variables, with R^2^ = 0.857; and yield quality exhibited R^2^= 0.799, indicating that microbial diversity, soil quality, and photosynthesis collectively explained nearly 80% of its variation (Figure 4).

These findings collectively underscore the ecological and functional centrality of microbial communities in shaping soil quality and crop productivity. While photosynthetic rate and vegetative growth are necessary physiological processes, they were not sufficient predictors of yield under the tested fertilization regimes. Instead, belowground biotic and abiotic interactions—especially those modulated by microbial diversity—play a dominant role in driving agricultural output and system resilience.

## 3. Discussion

This study provided a comprehensive evaluation of the impact of different types and application rates of organic fertilizers on soil physicochemical properties, microbial community structure, and tomato growth in a controlled greenhouse environment. The results highlighted the complex interactions between soil quality, rhizosphere microbial dynamics, and plant performance, emphasizing that fertilization practices can significantly influence agroecosystem functioning by altering soil nutrient availability and the rhizosphere microbiome.

To link nutrient inputs with plant performance, we compared the labeled dry-basis compositions with the as-applied (fresh-weight) rates. The BOF treatments supplied more nitrogen per hectare than BCF or BMF, and the largest increases in photosynthetic traits and yield were observed under the higher-rate BOF treatments. This pattern suggests that N availability was the predominant agronomic driver. Concurrent changes in soil properties (total N, Olsen-P, available K, and organic matter) reflect how inputs moved through and were processed by the soil. Within our conceptual framework, fertilizer type × rate determines nutrient delivery, and the soil mediates downstream effects on rhizosphere microbial structure and plant performance.

The application of high-dose bio-organic fertilizer (BOF4) resulted in a significant increase in microbial species richness, as indicated by elevated OTU and Chao1 indices. This suggested that nutrient-rich organic inputs fostered microbial biodiversity, a key factor in sustaining soil fertility and enhancing plant resilience. Notably, the Simpson index revealed that higher fertilization levels led to microbial dominance, potentially compromising community evenness. This finding was consistent with previous studies, which reported that organic amendments selectively enrich certain microbial taxa, often at the expense of others, thereby disrupting microbial equilibrium [18,19].

A particularly significant observation was the marked increase in the relative abundance of Proteobacteria under the BOF4 treatment. This phylum is well known for its essential roles in organic matter decomposition, nitrogen fixation, and plant pathogen suppression [19]. The selective enrichment of Proteobacteria at higher fertilization levels suggested that organic fertilizers promote the proliferation of functionally beneficial microbial taxa, thereby facilitating nutrient cycling and enhancing crop health. These results aligned with previous studies reporting that organic amendments stimulate the abundance of key microbial groups crucial for soil biogeochemical processes [20].

In addition to shifts in microbial communities, high-dose organic fertilization significantly increased soil organic matter (OM), total nitrogen (TN), available phosphorus (AP), and available potassium (AK), all of which were positively correlated with the abundance of Proteobacteria. These relationships suggested that microbial communities not only responded to nutrient enrichment but also actively mediated soil nutrient retention and transformation, reinforcing the critical role of microbial diversity in driving soil fertility and crop yield formation.

Beyond taxonomic composition, fertilization also altered the topology of microbial co-occurrence networks. Low-dose treatments were associated with higher clustering coefficients, indicating enhanced microbial cooperation and community stability. This supports the view that moderate nutrient availability fosters cooperative microbial associations and community stability, which are important for maintaining soil health. In contrast, high-dose treatments increased network complexity, as evidenced by a greater number of nodes, but concurrently reduced clustering coefficients. These changes suggested that while increased nutrient levels promoted microbial diversity, they also intensified interspecies competition, thereby reducing the structural cohesion of the microbial network. This pattern aligned with previous studies indicating that nutrient over-enrichment disrupts microbial interaction networks and weakens ecological stability [18,19]. Our findings implied that moderate application rates—particularly BOF2—optimized the balance between diversity and stability, enhancing microbial functionality without destabilizing community structure.

The model revealed that soil nutrient status (notably total N) and microbial diversity together structured the downstream responses, with the soil block serving as the proximal mediator between fertilization and plant traits. To further elucidate the mechanisms linking microbial diversity, soil function, and crop outcomes, we employed Partial Least Squares Path Modeling (PLS-PM). The model revealed that microbial diversity exerted the strongest direct effect on soil properties, reinforcing its central role in regulating soil nutrient availability and structure [20,21]. Improved soil properties, in turn, significantly enhanced plant growth and photosynthetic efficiency, highlighting the indirect pathways through which microbial diversity influences aboveground physiological processes. Accordingly, the stronger BOF responses are consistent with its higher N input per hectare, while paths are interpreted as associations rather than proof of direct causation.

Importantly, physiological parameters such as net photosynthetic rate (Pn), stomatal conductance (gs), and transpiration rate (Tr) showed positive associations with tomato yield and fruit quality. However, the PLS-PM results indicated that their direct effects on yield were weak and statistically insignificant. This discrepancy suggested that while photosynthetic efficiency contributed to plant growth, it was not the sole determinant of yield formation under organic fertilization. Instead, belowground interactions—particularly those mediated by microbial diversity and soil nutrient status—emerged as the dominant drivers of crop productivity. These findings underscored the quantitative relationship between physiological parameters and yield, while also explaining why their direct effects appeared limited when analyzed in the structural model.

This interpretation was further supported by Mantel’s test, which revealed strong positive correlations of OM, AP, AK, and TN with both yield and sugar content. In contrast, soil pH showed a significant negative correlation with AP and yield, indicating that soil acidification might have hindered phosphorus availability and reduced yield potential [22,23].

Future equal-N comparisons across fertilizer types would further disentangle type-specific effects from nutrient supply, complementing the system-level evidence reported here. While this study provided valuable insights into the short-term effects of organic fertilization, future research should focus on long-term field trials to assess the cumulative effects of organic fertilizers on soil health, microbial succession, and crop productivity. Moreover, integrating molecular approaches such as metagenomics and metabolomics will be crucial in uncovering the root–microbe feedback mechanisms that govern sustainable soil management.

## 4. Materials and Methods

### 4.1. Experimental Site Overview and Experimental Design

#### 4.1.1. Experimental Site and Greenhouse Overview

The experiment was conducted in a solar greenhouse located in Baoheshao Town, Saihan District, Hohhot, Inner Mongolia (40°51′ N, 111°41′ E; elevation 1060 m). The greenhouse was built in 2015, with an area of 1000 m^2^, and was equipped with automatic lift thermal curtains, temperature and humidity monitoring systems, and a drip irrigation system. The indoor terrain was flat, and the soil was a sandy loam classified as undisturbed native soil, which had not been cultivated or fertilized prior to the tomato experiment since the greenhouse was constructed. This soil therefore provided a reliable baseline for evaluating fertilizer effects. The average annual temperature of the surrounding area was 6.2 °C, with an average annual sunshine duration of 1600 h. For environmental characterization, we relied on in-greenhouse measurements rather than outdoor sunshine hours. During the experiment, the greenhouse internal temperature was maintained at 25–28 °C during the day and 12–18 °C at night by the automated regulation system to ensure a stable experimental environment. At canopy height, simultaneous indoor and outdoor photosynthetically active radiation (PAR) was logged at 15 min intervals during 10:00–12:00 on clear days; canopy light transmission was calculated as 100 × (PAR_in/PAR_out) after excluding shading-curtain periods. During the trial, the midday canopy light transmission was approximately 55–65%, and the indoor relative humidity (RH) was maintained within 60–70%. All plots shared the same greenhouse compartment, ensuring identical environmental conditions across treatments. The baseline nutrient and physicochemical properties of the soil (control group) were as follows: pH 7.2 ± 0.3, organic matter 21.7 ± 0.9 g/kg, total nitrogen 0.17 ± 0.008 mg/kg, available phosphorus 135.7± 0.5 mg/kg, and available potassium 225.0 ± 0.6 mg/kg.

#### 4.1.2. Experimental Materials

Tomato plants (cultivar ‘Jinuobili’, Wuhan Chuwei Biotechnology Co., Ltd., Wuhan, China) were used in the experiment. ‘Jinuobili’ is an indeterminate growth type tomato cultivar with individual fruit weighing over 250 g. The fruits are high-round in shape, combining firmness with good eating quality, and are well suited for storage and transportation. All organic fertilizers were produced by Inner Mongolia Dongbao Datian Biotechnology Co., Ltd., [Hohhot], China For the exact batches used, the product label/manufacturer’s certificate of analysis indicates a moisture content of 20–30% for all three products (BCF, BMF, and BOF) and a pH of 5.5–8.5. These products complied with the People’s Republic of China agricultural industry standard NY/T 525-2021 (Organic Fertilizer) [24]. The organic fertilizers tested included:

Bone Calcium Organic Fertilizer (BCF): A high-calcium bone powder substrate, fermented with microorganisms, containing organic matter ≥ 45% and total nutrients (N + P_2_O_5_ + K_2_O) ≥ 5.0% (dry basis N 1.2%, P_2_O_5_ 3.0%, K_2_O 0.8%; total 5.0%).

Bone Mud Organic Fertilizer (BMF): A fermented organic amendment derived from livestock bone residues and processing by-products (bone mud), rich in moisture and organic matter, with organic matter ≥ 30% and total nutrients (N + P_2_O_5_ + K_2_O) ≥ 4.0% (dry basis N 1.0%, P_2_O_5_2.2%, K_2_O 0.8%; total 4.0%). This material is distinct from bone meal, as it is processed through controlled microbial fermentation to ensure safety and nutrient stability before use. For nutrient accounting in this study, we used a dry-basis total nutrients (N + P_2_O_5_ + K_2_O) of 4.0% (composition: N 1.0%, P_2_O_5_ 2.2%, K_2_O 0.8%).

Biological Organic Fertilizer (BOF): A powder carrier inoculated with a beneficial microbial consortium (BMC)—a mixed culture of functional microbes including photosynthetic bacteria, lactic acid bacteria, and yeasts; effective viable bacteria ≥ 0.2 billion/g, organic matter ≥ 65%, and total nutrients ≥ 8.0% (dry basis N 3.5%, P_2_O_5_ 3.0%, K_2_O 1.5%; total 8.0%).

#### 4.1.3. Experimental Design

The experiment included three treatment groups: Bone Calcium Organic Fertilizer (BCF), Bone Mud Organic Fertilizer (BMF), and Biological Organic Fertilizer (BOF), as well as a control group with no fertilization (Control). In the study region, mineral fertilizers are not routinely used as a baseline practice in greenhouse tomato production, where organic amendments or unfertilized baseline conditions are commonly adopted to maintain soil health, reduce chemical inputs, and support the production of premium-quality, eco-friendly vegetables. Therefore, the unfertilized control was selected to reflect prevailing production practices and to provide a consistent baseline for evaluating the relative effects of different organic fertilizers and application rates. Each treatment included four fertilization gradients (Table 1) [25,26]. A total of 39 plots were used, with three biological replicates for each treatment, and each plot was 25 m^2^. Before planting, the soil was plowed, and the corresponding fertilizers were evenly applied to the fertilized plots. Fertilizers were broadcast uniformly and immediately incorporated into the topsoil (0–15 cm) using a rotary tiller within 2 h of application; the control plots received identical tillage without fertilizer. Fertilizers were applied as a single basal dose 10 days before transplanting; no topdressing was applied. Transplanting began in July 2022. Seedling status at transplanting. Plants were nursery-raised (pre-germinated) seedlings and were 13.0 cm tall at transplanting. Furrows were made before planting (row spacing 1.25 m), a drip irrigation system was installed, and plant spacing was set at 0.45 m. During the experiment, standard greenhouse tomato practices were followed for irrigation and fertilization management, pest and disease control, and routine maintenance to ensure consistency across all plots. Final harvest and experiment termination occurred at 98 days after planting (98 DAP).

### 4.2. Measurement Indicators and Methods

#### 4.2.1. Measurement of Plant Growth and Physiological Indicators

During the tomato growth period, plant growth and photosynthetic physiological indicators were measured. Fifteen tomato plants with uniform growth were randomly selected from each plot for fixed observation, resulting in a total of 45 plants per treatment (three replicates per treatment). From planting to flowering, plant height and stem diameter were measured every 14 days (five measurements in total). Plant height was measured as the natural height of the plant (from the soil surface to the tip), and stem diameter was measured 3 cm above the soil surface using a digital electronic caliper (Mitutoyo Corp., Kawasaki, Japan). During the flowering, fruit expansion, and harvest stages, stomatal conductance (gs), net photosynthetic rate (Pn), and transpiration rate (Tr) were measured using a portable LI-6400 photosynthesis system (LI-COR Biosciences, Lincoln, NE, USA). For each fixed observation plant, three fully expanded healthy functional leaves were selected from the top down to ensure consistent leaf position, uniform light exposure, and absence of pest or disease damage.

#### 4.2.2. Yield and Soluble Solids Content (SSC) Measurement

At harvest, yield and soluble solids content (SSC) were measured weekly. For yield determination, all mature fruits from each plot were harvested and weighed, and the yield per hectare was calculated based on the plot area. For SSC determination, 15 random fruits from each plot were selected, deseeded, and homogenized using a laboratory homogenizer (IKA T18 Ultra-Turrax, Staufen, Germany). The homogenate was filtered through a fine mesh to remove solids, and the juice was analyzed using a digital refractometer (PAL-1, Atago Co., Ltd., Tokyo, Japan) based on the standard Brix method, with SSC expressed as °Brix for each plot [27].

#### 4.2.3. Measurement of Soil Physicochemical Properties

Starting 28 days after planting (DAP), soil was sampled every 14 days from the 0–20 cm layer using a five-point composite per plot. Soil moisture was tracked at each campaign with a handheld meter; no continuous sensor-based monitoring was used. At 70 DAP, soil from the 0–20 cm layer was collected by the same five-point method for physicochemical analyses, concurrently with rhizosphere sampling for microbiological analyses, so that measurements were temporally aligned. The samples were air-dried, ground (passed through a 2 mm sieve), and then analyzed in the laboratory. Soil organic matter was determined using the K_2_Cr_2_O_7_ volumetric method (analytical-grade reagents; Sigma-Aldrich LLC, St. Louis, MO, USA); total nitrogen was measured with an elemental analyzer (vario MACRO cube; Elementar Analysensysteme GmbH, Langenselbold, Germany). with 100–110 mg of soil sample per replicate (three replicates per treatment). Available potassium was extracted using a 1:5 soil-to-water ratio, while exchangeable potassium was extracted with neutral ammonium acetate (1 M NH_4_OAc, pH 7.0) and determined using a flame photometer (Model 410, Sherwood Scientific Ltd., Cambridge, UK). pH was measured using a 1:5 and 1:2.5 soil-to-water ratio extraction with a Hanna multi-parameter analyzer Hi-4522 (Italy). Available phosphorus (Olsen-P; 0.5 mol L^−1^ NaHCO_3_, pH 8.5) was determined colorimetrically (molybdenum-blue) at 880 nm using a UV–Vis spectrophotometer (UV-2600, Shimadzu Corporation, Kyoto, Japan) [28].

#### 4.2.4. Soil Microbial Analysis

At 70 days after planting (70 DAP), rhizosphere soil (0–20 cm) was collected using a five-point composite sampling method, with three composite samples per replicate plot (nine samples per treatment). After homogenization, soil samples were placed into sterile 15 mL centrifuge tubes, immediately stored on dry ice, and transported to the laboratory, where they were preserved at −80 °C until analysis. Genomic DNA was extracted from 0.5 g of each sample using a magnetic bead-based soil genomic DNA extraction kit (Mag-Bind Soil DNA Kit, Omega Bio-Tek, Norcross, GA, USA) following the manufacturer’s protocol. DNA purity and concentration were determined by 1% agarose gel electrophoresis and quantified using a NanoDrop spectrophotometer (Thermo Fisher Scientific, Waltham, MA, USA). DNA was diluted to 1 ng/μL using sterile water for subsequent analyses. Soil 16S rRNA gene amplicon sequencing targeting the V4 region was performed by Novogene Co., Ltd. (Beijing, China) on an Illumina MiSeq platform, as described in previous studies [29,30,31]. Sequencing data were processed using Python 3.6.13 for demultiplexing and analyzed using QIIME2 2022.2 and R 4.0.3 for microbial taxonomic identification, alpha and beta diversity assessment, and co-occurrence network construction. These analyses focused on microbial community composition and relative abundance rather than absolute quantification.

### 4.3. Data Analysis

Experimental data were organized using Excel and analyzed using R statistical software. Ecological network analysis was performed using Gephi (version 0.9.7) to build the node and edge database, based on robust correlations with a correlation coefficient |r| > 0.6 and *p* < 0.001 [32,33]. Network analysis was conducted using R’s “vegan” and “igraph” packages, with visualization in Gephi [34]. Co-occurrence relationships, network connectivity, and complexity were measured by average degree and path length. Mantel’s test was used to assess the correlations between microbial communities, plant traits, and soil physicochemical properties, with calculation performed using R’s “linkET” and “dplyr” packages. Partial least squares path modeling (PLS-PM) was carried out using R’s “plspm” package [35]. Differences among treatments were evaluated using one-way ANOVA, followed by Tukey’s HSD test (*p* < 0.05) for post hoc comparisons, ensuring robust assessment of treatment effects across plant, soil, and microbial variables. All statistical analyses were performed in R (version 4.0.3).

## 5. Conclusions

Under greenhouse conditions on sandy loam, fertilizer type and rate jointly influenced tomato physiology, yield, soil properties, and the rhizosphere microbiome. Higher application rates of N-rich bio-derived organic fertilizers were associated with greater photosynthetic performance and yield, alongside increases in soil organic matter and total nitrogen. Network and path analyses indicated associations linking improved soil properties to higher microbial diversity/connectivity and, in turn, to crop performance; these reflect indirect relationships rather than causation and may vary by soil context. For N-poor but P/K-rich soils similar to ours, total organic inputs of 30,000–45,000 kg·ha^−1^ appeared favorable; however, we recommend soil-test–guided management and caution against extrapolation to systems with different texture, nutrient status, or mineral-fertilizer baselines.

## Figures and Tables

**Table 1:** Experimental treatment design.

Treatment	Fertilizer Application Rate (Wet Weight, kg·ha^−1^)	Treatment Code
Bone-calcium organic fertilizer (BCF)	7500	BCF1
	15,000	BCF2
	30,000	BCF3
	45,000	BCF4
Bone-mud organic fertilizer (BMF)	7500	BMF1
	15,000	BMF2
	30,000	BMF3
	45,000	BMF4
Bio-organic fertilizer (BOF)	7500	BOF1
	15,000	BOF2
	30,000	BOF3
	45,000	BOF4
Control	0	Control

## Data Availability

The raw data supporting the conclusions of this article are available from the corresponding author on reasonable request.

## Supplementary Materials

The following supporting information can be downloaded at: https://www.mdpi.com/article/10.3390/plants14213333/s1, Figure S1: Tomato plant height and stem diameter under different fertilization treatments. (a) Tomato plant height under different fertilization treatments. (b) Tomato stem diameter under different fertilization treatments; Table S1. Photosynthetic gas exchange parameters of greenhouse tomatoes under different fertilization treatments. Table S2. Tomato Yield and Quality Analysis. Table S3: Soil moisture content (%) under different fertilization treatments; Table S4: Changes in soil pH and fertility under different fertilization treatments for greenhouse tomatoes; Table S5: Topological parameter calculation for soil microbial co-occurrence networks. Table S6. Topological Parameter Calculation.
