# Supplementary material for: Effects of Organic Fertilizer Type and Application Rate on Soil–Microbe Interactions, Yield, and Quality of Greenhouse Tomato
Source: Plants (Basel). 2025 Oct 31;14(21):3333. doi: 10.3390/plants14213333 (PMC12608545; doi:10.3390/plants14213333)
Supplement: Supplementary file 1 [file plants-14-03333-s001.zip › plants-3858159-supplementary.pdf]

Effects of Organic Fertilizer Type and Application Rate on Soil–Microbe Interactions, Yield, and Quality of Greenhouse Tomato

Supplementary Materials

Jingshi Lu [1,†], Xiaoming Zhang [1,†], Yingtong Mu [1], Jiahui Gao [2], Fengyan Yi [3], Ping Wang [1], Doudou Jin [1], Fang Tang [1,*] and Wenqiang Fan [1,*]

[1] Key Laboratory of Grassland Resources, College of Grassland Science, Mongolian and Chinese Medicinal Plant Germplasm Breeding Engineering Technology Research Center, Inner Mongolia Agricultural University, Ministry of Education, Hohhot 010011, China; jingshilu37@gmail.com (J.L.); bagenna123@aliyun.com (X.Z.); myt100862@outlook.com (Y.M.); 19819262059@163.com (P.W.); chinajddimau@163.com (D.J.)

[2] Inner Mongolia Forestry and Grassland Monitoring and Planning Institute, Hohhot 010011, China; 13474710702@163.com

[3] Inner Mongolia Academy of Agricultural and Animal Husbandry Sciences, Hohhot 010031, China; yifengyanonly88@126.com

* Correspondence: tangfang@imau.edu.cn (F.T.); fanwenqiang@imau.edu.cn (W.F.)

† These authors contributed equally to this work.

Table S1. Photosynthetic gas exchange parameters of greenhouse tomatoes under different fertilization treatments

Treatment	Pn（mmol·m^{-2} s^{-1}）			gs（mol·m^{-2} s^{-1}）			Tr（mmol·m^{-2} s^{-1}）		
	Flowering stage	Fruit expansion stage	Harvest stage	Flowering stage	Fruit expansion stage	Harvest stage	Flowering stage	Fruit expansion stage	Harvest stage
Control	27.0±0.74f	25.2±1.29e	15.6±0.64f	0.30±0.03i	0.34±0.006e	0.25±0.03e	15.4±0.1h	10.1±0.0e	12.9±0.02ef
BCF1	30.2±0.82de	25.5±0.20e	16.6±0.20de	0.35±0.02efgh	0.39±0.02cd	0.29±0.02c	16.7±0.1efg	12.0±1.5d	13.1±0.6ef
BCF2	31.9±0.61bc	29.5±1.16bc	17.6±0.28bc	0.37±0.01defg	0.42±0.006bc	0.29±0.01cd	17.4±0.6cde	13.1±0.7bc	13.2±0.0de
BCF3	32.3±0.21bc	29.9±1.47bc	17.9±0.73bc	0.38±0.006cd	0.44±0.02b	0.30±0.01bc	17.7±0.1cd	13.2±0.6abc	13.5±0.0bc
BCF4	33.3±0.26c	30.7±0.82b	18.4±0.41b	0.42±0.02b	0.49±0.02a	0.32±0.01ab	18.9±0.6a	14.3±1.0a	13.6±0.0ab
BMF1	27.3±0.31f	25.9±2.24e	16.0±1.11ef	0.32±0.02hi	0.39±0.01d	0.27±0.02de	16.0±0.6gh	11.6±0.0d	12.9±0.0ef
BMF2	27.5±2.70f	26.7±0.46de	15.7±0.10f	0.34±0.02fgh	0.40±0.01cd	0.28±0.006cd	16.7±0.1efg	12.6±0.6cd	13.2±0.0de
BMF3	29.5±0.32e	29.2±0.37bc	17.6±0.18bc	0.37±0.02defg	0.44±0.03b	0.29±0.02c	17.7±0.02cd	13.2±0.6abc	13.4±0.0bcd
BMF4	32.0±0.89bc	30.0±0.32b	18.0±0.16bc	0.41±0.006bc	0.48±0.006a	0.30±0.01bc	17.9±1.1bc	13.9±0.6ab	13.5±0.2bc
BOF	31.5±0.3	28.3±0	17.2±0	0.34±0.	0.40±0	0.30±0	17.0±0.	12.7±0.8	13.4±0.0

1	2cd	.66cd	.36cd	01fgh	.01cd	.02bc	6def	cd	bcd
BOF2	31.9±0.62bc	29.2±0.58bc	17.8±0.58bc	0.36±0.04defg	0.43±0.02b	0.30±0.02bc	17.7±0.1cd	13.2±0.6abc	13.4±0.0bcd
BOF3	32.8±0.54bc	30.2±0.06b	18.1±0.04b	0.38±0.01cd	0.47±0.04a	0.31±0.01ab	18.6±0.0ab	13.3±0.7abc	13.6±0.0ab
BOF4	34.9±1.63a	32.8±1.56a	19.4±0.78a	0.45±0.04a	0.51±0.01a	0.33±0.006a	19.2±0.6a	14.1±0.5ab	13.8±0.1a

Note: Different superscript letters indicate significant differences among treatments (one-way ANOVA, Tukey's HSD, $P < 0.05$); the same letter indicates no significant difference.Abbreviations. Pn, net photosynthetic rate (μmol $CO_2 \cdot m^{-2} \cdot s^{-1}$); gs, stomatal conductance (mol $H_2O \cdot m^{-2} \cdot s^{-1}$); Tr, transpiration rate (mmol $H_2O \cdot m^{-2} \cdot s^{-1}$); OM, organic matter; TN, total nitrogen; AP, available phosphorus (Olsen-P); AK, available potassium; EC, electrical conductivity; DAP, days after planting.Treatment codes. Control, no fertilization; BCF1–BCF4, bone calcium organic fertilizer at 7500, 15000, 30000, 45000 $kg \cdot ha^{-1}$; BMF1–BMF4, bone mud organic fertilizer at 7500, 15000, 30000, 45000 $kg \cdot ha^{-1}$; BOF1–BOF4, bio-organic fertilizer at 7500, 15000, 30000, 45000 $kg \cdot ha^{-1}$.

Table S2. Tomato yield and quality analysis

Note: Different superscript letters indicate significant differences among treatments (one-way ANOVA,

Treatment	Single-plant yield (kg)	Yield $kg \cdot ha^{-1}$	Soluble solids content (SSC °Brix)
Control	2.0±0.1h	34496 ± 1056e	5.2±0.2e
BCF1	2.2±0.0g	37840 ± 704cde	6.4±0.1d
BCF2	2.6±0.1d	45760 ± 2112abcd	6.6±0.1cd
BCF3	2.7±0.1c	47908 ± 1936abc	6.6±0.1cd
BCF4	2.7±0.1c	48048 ± 2364abc	7.1±0.5a
BMF1	2.0±0.2h	35728 ± 2640de	6.5±0.5cd
BMF2	2.3±0.2f	40702 ± 3072bcde	6.4±0.2d
BMF3	2.3±0.4f	40832 ± 3216bcde	6.6±0.2cd
BMF4	2.8±0.2b	49984 ± 2640ab	7.0±0.2ab
BOF1	2.2±0.1g	39248 ± 1760cde	6.5±0.3cd
BOF2	2.5±0.2e	44704 ± 3696abcde	6.4±0.1d
BOF3	2.6±0.3d	45584 ± 4104abcd	6.8±0.2bc
BOF4	3.0±0.3a	52096 ± 4928a	7.2±0.3a

Tukey's HSD, $P < 0.05$); the same letter indicates no significant difference. Abbreviations and treatment codes are as in Table S1.

Table S3. Soil moisture content (%) under different fertilization treatments

Treatment	28d	42d	56d	70d	84d	98d
Control	27.4±1.3c	34.5±0.4b	41.9±0.6c	31.7±0.4bc	26.2±1.1b	25.6±0.4d
BCF1	27.0±1.3c	32.8±1.7bcd	40.9±1.6cde	31.7±0.8bc	26.7±1.0b	27.4±1.1bcd

BCF2	31.0±0.4ab	40.0±0.2a	46.4±1.3b	37.8±0.1a	32.8±1.3a	33.5±2.0a
BCF3	26.9±0.3c	32.6±0.5bcd	41.1±1.0cd	32.6±1.6bc	25.7±0.7bc	26.3±1.1bcd
BCF4	24.3±0.7d	31.3±1.8cd	41.8±0.9c	32.8±0.9b	23.5±1.4bc	27.3±1.1bcd
BMF1	23.2±0.4de	31.1±2.4d	40.1±2.2de	31.1±1.8bc	23.4±0.8bc	26.9±0.9bcd
BMF2	22.9±2.1de	32.8±1.4bcd	39.4±1.1e	31.9±0.8bc	27.0±4.7b	28.0±1.0bc
BMF3	32.1±1.8a	40.6±1.0a	47.2±0.6ab	38.2±1.1a	31.5±1.2a	32.6±1.6a
BMF4	26.9±0.2c	32.7±0.5bcd	40.8±0.4cde	32.2±0.8bc	26.2±1.7b	26.8±1.0bcd
BOF1	29.5±0.1b	39.5±0.7a	48.1±0.5a	30.9±1.2bc	32.6±0.5a	33.3±2.1a
BOF2	26.5±0.6c	32.1±0.6cd	40.3±1.1cde	31.5±1.8bc	25.4±1.1bc	27.0±2.4bcd
BOF3	19.5±1.6f	31.0±1.1d	41.8±0.6c	32.4±2.2bc	26.0±1.4b	25.9±0.5cd
BOF4	21.5±1.9e	33.1±1.8bc	41.4±0.7cd	30.8±0.6cd	22.2±5.6c	28.2±0.6b

Note: Abbreviations and treatment codes are as in Table S1.

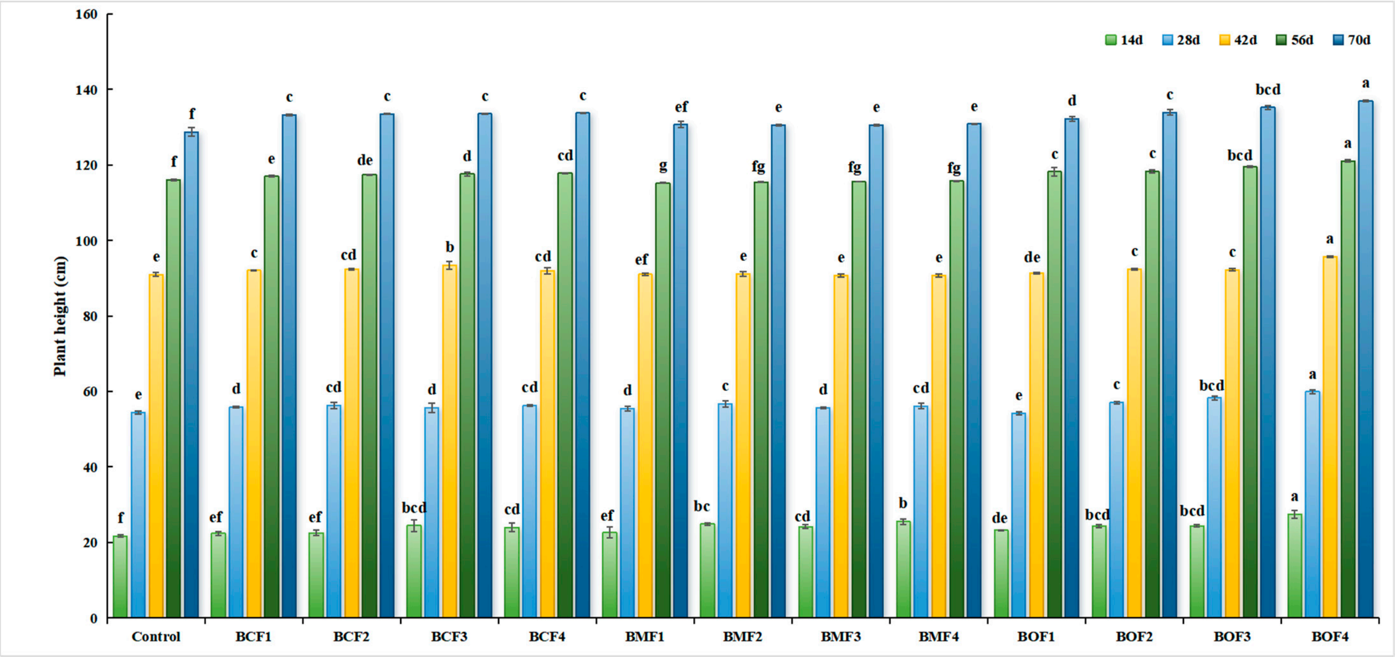

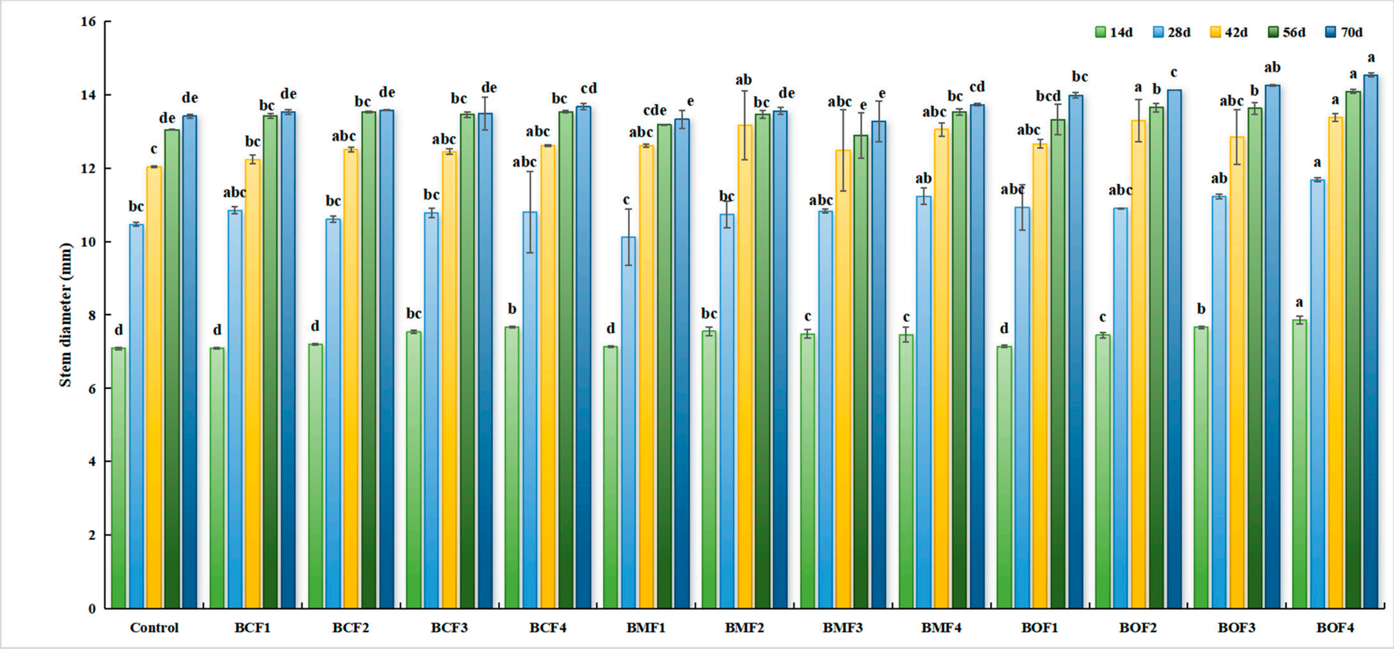

Figure S1. Tomato plant height and stem diameter under different fertilization treatments. (a)Tomato plant height under different fertilization treatments. (b)Tomato stem diameter under different fertilization treatments.

Table S4. Soil pH and fertility indices at the microbiological sampling time (70 DAP) under different fertilization treatments.

Treatment	pH	Organic matter content (g/kg)	Total nitrogen content (mg/kg)	Available phosphorus content (mg/kg)	Available potassium content (mg/kg)
Control	7.2±0.3a	21.7±0.9h	0.17±0.008f	135.7±0.5h	225.0±0.6l
BCF1	6.8±0.1bc	26.6±2.2f	0.17±0.006f	143.7±0.6fg	249.1±0.7j
BCF2	6.6±0.0bcd	26.9±1.3e	0.20±0.002e	145.6±0.7e	262.0±0.8h
BCF3	6.4±0.0de	28.3±1.5c	0.23±0.006d	149.8±0.4c	275.9±0.9d
BCF4	6.1±0.0f	29.2±1.5b	0.28±0.008b	152.1±0.2b	285.6±1.0b
BMF1	6.9±0.1b	25.5±1.5g	0.17±0.007f	133.9±1.3i	247.4±0.2k
BMF2	6.7±0.0bc	27.7±4.8de	0.20±0.009e	143.3±0.6g	263.6±0.6g
BMF3	6.5±0.1de	27.5±2.9de	0.23±0.004d	146.5±0.3de	268.4±1.1f
BMF4	6.3±0.1ef	28.5±1.1c	0.25±0.013c	150.8±0.6c	279.5±1.4c
BOF1	6.7±0.0bc	26.4±3.7f	0.18±0.004f	144.4±1.1f	252.1±0.6i
BOF2	6.6±0.1bcd	27.2±5.2e	0.22±0.006d	146.9±0.8d	270.4±0.9e
BOF3	6.3±0.0ef	29.0±2.7b	0.25±0.005c	150.5±0.3c	279.2±0.2c
BOF4	6.0±0.0f	30.9±1.3a	0.31±0.006a	155.0±0.9a	287.7±1.2a

Note: Abbreviations and treatment codes follow Table S1. pH denotes the measure of soil acidity/alkalinity (dimensionless).

Table S5. Overview of raw sequencing reads, quality control, and effective data.

Sample	RawPE	Combined	Qualified	Nochime	Base(nt)	Avglen(nt)	GC（%）	Q20（%）	Q30（%）	Effective（%）
Control	137215	136412	136173	127033	32164324	253.2	56.1	99.5	98.1	92.6
Control	132985	132268	132045	123175	31190382	253.2	56	99.4	97.7	92.6
Control	135909	135156	134917	125397	31752058	253.2	55.7	99.4	97.9	92.3
BCF1	115645	115011	114824	106587	26986861	253.2	55.7	99.5	98.0	92.2
BCF1	134788	134013	133827	124192	31445349	253.2	56.0	99.6	98.2	92.1
BCF1	136044	135380	135222	125164	31694431	253.2	56.0	99.5	98.0	92.0
BCF2	137098	136396	136131	127690	32330954	253.2	55.8	99.4	97.9	93.1
BCF2	138247	137458	137174	128267	32476111	253.2	55.7	99.4	97.7	92.8
BCF2	134240	133525	133230	123984	31390224	253.2	55.6	99.4	97.7	92.4
BCF3	136918	136169	135885	126597	32057077	253.2	56.3	99.4	97.8	92.5
BCF3	133681	132913	132640	123628	31301905	253.2	56.1	99.3	97.6	92.5
BCF3	144542	143742	143418	134737	34120718	253.2	56.0	99.4	97.8	93.2
BCF4	123530	122918	122670	115066	29136291	253.2	55.9	99.4	97.8	93.2
BCF4	132758	131993	131724	122859	31112222	253.2	56.3	99.5	98.0	92.5
BCF4	96266	95765	95598	90857	23010299	253.3	56.2	99.4	97.8	94.4
BMF1	124097	123392	123097	116009	29374174	253.2	56.2	99.4	97.8	93.5
BMF1	136084	135234	134990	126016	31908887	253.2	56.2	99.5	98.1	92.6
BMF1	136136	135419	135143	126631	32067487	253.2	55.9	99.5	98.0	93.0
BMF2	136694	135815	135551	126148	31944102	253.2	56.3	99.5	98.1	92.3
BMF2	135236	134450	134161	124592	31552757	253.3	56.3	99.4	97.7	92.1

BMF2	132987	132167	131901	122267	30965617	253.3	56.0	99.4	97.9	91.9
BMF3	132439	131752	131491	122026	30903555	253.3	56.1	99.4	97.9	92.1
BMF3	135978	134977	134713	125802	31864168	253.3	56.4	99.5	98.1	92.52
BMF3	111439	110899	110742	103381	26181427	253.3	56.2	99.5	97.9	92.8
BMF4	135593	134841	134548	125346	31737996	253.2	56.0	99.4	97.8	92.4
BMF4	132068	131060	130822	121520	30771882	253.2	56.2	99.6	98.1	92.0
BMF4	132441	131710	131451	121931	30871106	253.2	55.6	99.4	97.9	92.1
BOF1	133947	133239	132946	123718	31325449	253.2	56.3	99.4	97.9	92.4
BOF1	132455	131707	131443	124232	31464031	253.3	56.4	99.5	97.9	93.8
BOF1	132403	131619	131362	121878	30864059	253.2	56.0	99.4	97.9	92.1
BOF2	112847	112260	112005	104055	26347123	253.2	55.9	99.5	98.0	92.2
BOF2	134342	133557	133284	124365	31493828	253.2	56.2	99.5	98.1	92.6
BOF2	94051	93622	93442	88020	22291575	253.3	56.0	99.5	98.0	93.6
BOF3	133410	132670	132355	124437	31510767	253.2	55.9	99.4	97.9	93.3
BOF3	138528	137238	136982	130254	32990869	253.3	55.9	99.5	98.0	94.0
BOF3	135489	134747	134485	125484	31782133	253.3	56.0	99.4	97.9	92.6
BOF4	91719	91243	91074	83869	21239317	253.2	55.7	99.4	97.9	91.4
BOF4	131853	131153	130869	121458	30747164	253.2	55.1	99.4	97.7	92.1
BOF4	137849	137237	136994	127725	32339518	253.2	55.5	99.4	97.9	92.7

Table S6. Topological parameter calculation

Treatment	Density	Diameter	Average_Degree	Clustering_Coefficient	Modularity	Avg_Path_Length	Node_Count	Edge_Count
Control	0.049	2	12.1	1	0.86	2	249	1503
BCF1	0.049	2	12.0	1	0.85	2	246	1473
BCF2	0.061	2	16.3	1	0.80	2	271	2214
BCF3	0.044	2	11.4	1	0.91	2	258	1468
BCF4	0.046	2	11.2	1	0.917	2	247	1388
BMF1	0.061	2	17.5	1	0.75	2	287	2512
BMF2	0.044	2	11.4	1	0.91	2	262	1493
BMF3	0.040	2	9.8	1	0.92	2	248	1214
BMF4	0.051	2	13.4	1	0.88	2	265	1773
BOF1	0.045	2	12.0	1	0.92	2	269	1615
BOF2	0.045	2	10.9	1	0.92	2	243	1325
BOF3	0.046	2	13.2	1	0.90	2	288	1896
BOF4	0.047	2	12.1	1	0.88	2	258	1556

Note: Abbreviations and treatment codes are as in Table S1.
